# Efzofitimod for the Treatment of Pulmonary Sarcoidosis

**DOI:** 10.1016/j.chest.2022.10.037

**Published:** 2022-11-08

**Authors:** Daniel A. Culver, Shambhu Aryal, Joseph Barney, Connie C.W. Hsia, W. Ennis James, Lisa A. Maier, Lucian T. Marts, Ogugua Ndili Obi, Peter H.S. Sporn, Nadera J. Sweiss, Sanjay Shukla, Nelson Kinnersley, Gennyne Walker, Robert Baughman

**Affiliations:** a Cleveland Clinic, Cleveland, OH; b Advanced Lung Disease and Lung Transplant Program, Inova Fairfax Hospital, Falls Church, VA; c Department of Pulmonary and Critical Care Medicine, University of Alabama, Birmingham, AL; d Division of Pulmonary and Critical Care Medicine, Department of Internal Medicine, University of Texas Southwestern Medical Center, Dallas, TX; e Susan Pearlstine Sarcoidosis Center of Excellence, Pulmonary and Critical Care Medicine, Medical University of South Carolina, Charleston, SC; f Division of Environmental and Occupational Health Sciences, National Jewish Health; g Division of Pulmonary Sciences and Critical Care, Department of Medicine, School of Medicine, University of Colorado, Denver, CO; h Division of Pulmonary, Allergy, Critical Care, and Sleep Medicine, Department of Medicine, Emory University School of Medicine, Atlanta, GA; i Division of Pulmonary, Critical Care and Sleep Medicine, Department of Internal Medicine, Brody School of Medicine East Carolina University, Greenville, NC; j Division of Pulmonary and Critical Care Medicine, Department of Medicine, Northwestern University Feinberg School of Medicine, Chicago, IL; k Division of Rheumatology and Medical Director of the Arthritis Clinic, Chicago, IL; l Bernie Mac Sarcoidosis Translational Advanced Research Center, University of Illinois College of Medicine, Chicago, IL; m aTyr Pharma, Inc., San Diego, CA; n Octa Consulting Services, Ltd., Harpenden, England; o Department of Medicine, University of Cincinnati Medical Center, Cincinnati, OH

**Keywords:** ATYR1923, corticosteroids, efzofitimod, fatigue assessment scale, immunomodulator, lung function, neuropilin 2, pulmonary sarcoidosis, quality of life, steroid taper

## Abstract

**Background:**

Pulmonary sarcoidosis is characterized by the accumulation of immune cells that form granulomas affecting the lungs. Efzofitimod (ATYR1923), a novel immunomodulator, selectively binds neuropilin 2, which is upregulated on immune cells in response to lung inflammation.

**Research Question:**

What is the tolerability, safety, and effect on outcomes of efzofitimod in pulmonary sarcoidosis?

**Study Design And Methods:**

In this randomized, double-blind, placebo-controlled study evaluating multiple ascending doses of efzofitimod administered intravenously every 4 weeks for 24 weeks, randomized patients (2:1) underwent a steroid taper to 5 mg/d by week 8 or < 5 mg/d after week 16. The primary end point was the incidence of adverse events (AEs); secondary end points included steroid reduction, change in lung function, and patient-reported outcomes on health-related quality-of-life scales.

**Results:**

Thirty-seven patients received at least one dose of study medication. Efzofitimod was well tolerated at all doses, with no new or unexpected AEs and no dose-dependent AE incidence. Average daily steroid doses through end of study were 6.8 mg, 6.5 mg, and 5.6 mg for the 1 mg/kg, 3 mg/kg, and 5 mg/kg groups compared with 7.2 mg for placebo, resulting in a baseline-adjusted relative steroid reduction of 5%, 9%, and 22%, respectively. Clinically meaningful improvements were achieved across several patient-reported outcomes, several of which reached statistical significance in the 5 mg/kg dose arm. A dose-dependent but nonsignificant trend toward improved lung function also was observed for 3 and 5 mg/kg.

**Interpretation:**

Efzofitimod was safe and well tolerated and was associated with dose-dependent improvements of several clinically relevant end points compared with placebo. The results of this study support further evaluation of efzofitimod in pulmonary sarcoidosis.

**Trial Registry:**

ClinicalTrials.gov; No.: NCT03824392; URL: www.clinicaltrials.gov

## Graphical Abstract

**Figure:**
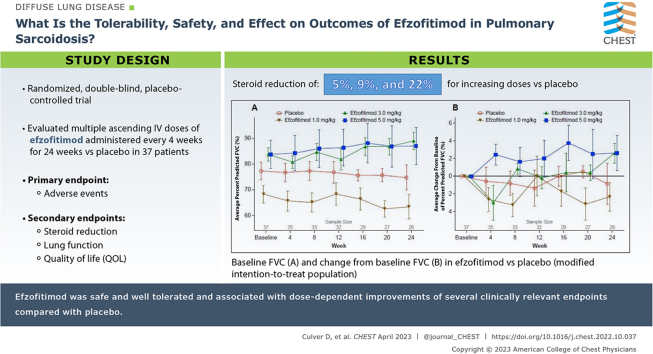

**Take-home Points**

**ResearchQuestion:** What is the tolerability of efzofitimod for pulmonary sarcoidosis, and can we discern any evidence of clinical efficacy to support a larger trial?

**Results**: No differences were found in adverse effects or tolerability between participants randomized to efzofitimod or placebo. Patient-reported outcomes improved in the higher-dose arms and positive trends for other end points were found.

**Interpretation:** Efzofitimod may be useful for pulmonary sarcoidosis. Larger studies are needed to confirm and extend these findings.

Sarcoidosis is a multisystem, granulomatous disorder that most commonly affects the lungs.^1^ Patients often demonstrate organ-specific symptoms such as dyspnea and cough, but also show a range of other disabling nonspecific symptoms (eg, fatigue) that have a major impact on quality of life (QOL). For patients with pulmonary sarcoidosis, the goal of treatment is to reduce the risk of death or permanent disability (danger) or to improve the patient’s QOL,^2^ while secondarily managing the inflammation that may lead to pulmonary fibrosis and irreversible loss of lung function.^3^^,^^4^ The consensus standard of care includes oral corticosteroids that act mainly by suppressing inflammatory genes.^1^^,^^5^ Although corticosteroid therapy has been shown to stabilize or improve the disease, long-term corticosteroid use is associated with significant side effects, including substantial weight gain, development of insulin resistance, risk of infection,^6^ and impaired QOL.^7^ Alternatives, such as immunosuppressive and cytotoxic agents (eg, methotrexate), can be used; however, these therapies also have significant side effects and toxicities.^8^ Hence, a need exists to find new and effective treatments for pulmonary sarcoidosis with fewer side effects and a positive impact on QOL.

Efzofitimod (ATYR1923) is a novel IV biological immunomodulator composed of a splice variant of histidyl-tRNA synthetase^9^^,^^10^ that encodes the immunomodulatory domain that binds to the neuropilin 2 receptor protein.^11^ Neuropilin 2 is a pleiotropic receptor^12^ that is upregulated on the surface of activated immune cells responsible for inflammation and granuloma formation in the lungs of patients with pulmonary sarcoidosis.^13^ Preclinical studies have shown that efzofitimod regulates immune responses^14^, ^15^, ^16^ and significantly reduces lung fibrosis and inflammation.^17^^,^^18^ Thus, efzofitimod may leverage a naturally occurring human immunomodulatory function to control or balance the human immune system therapeutically.

In healthy volunteers, single doses of efzofitimod (0.03-5 mg/kg) are well tolerated, with no significant safety concerns.^19^ Efzofitimod pharmacokinetics are dose proportional over the range of 0.03 to 5.0 mg/kg, with a mean half-life ranging from 167 to 242 h (7-10 days), supporting once every 4 weeks dosing.^19^ Herein, we present the primary clinical data from the first investigation of efzofitimod in patients with pulmonary sarcoidosis designed to evaluate the safety, tolerability, and preliminary efficacy in this patient population.

## Study Design and Methods

### Trial Design and Procedures

Patients were 18 to 75 years of age, had a diagnosis of pulmonary sarcoidosis for ≥ 6 months according to the 1999 American Thoracic Society standards,^20^ and showed evidence of parenchymal involvement. The full inclusion and exclusion criteria are provided in e-Appendix 1.

This was a randomized, double-blind, placebo-controlled multiple ascending dose study with three sequential dose cohorts with a 2:1 randomization (efzofitimod to placebo) in each cohort; the planned study size was 36 patients (ClinicalTrials.gov Identifier: NCT03824392). Patients receiving placebo from each of the three cohorts were pooled when comparing safety and efficacy between placebo and efzofitimod. The treatment period consisted of six IV administrations of study drug (efzofitimod or placebo) once every 4 weeks for a total of 20 weeks (at day 1 and weeks 4, 8, 12, 16, and 20), with the final study assessments conducted at week 24.

Safety and tolerability assessments consisted of evaluation of treatment-emergent adverse events (TEAEs), physical examinations, vital signs and temperature, 12-lead ECGs, pulse oximetry, weight, immunogenicity, and clinical laboratory tests. Assessment of daily corticosteroid dose over the study period (day 1-week 24) and the number of patients who achieved the targeted tapered dose of prednisone 5 mg/day (or equivalent) through week 24 also were recorded. Pulmonary function tests including the FVC % predicted and diffusing capacity of the lungs for carbon monoxide (Dlco) % predicted were also performed. Patient-reported QOL was assessed by the Sarcoidosis Assessment Tool (SAT), the King’s Sarcoidosis Questionnaire (KSQ), the Leicester Cough Questionnaire, the Fatigue Assessment Scale (FAS), and the Self-Administered Computerized Baseline and Transitional Dyspnea Indices. The schedule of assessments is presented in e-Table 1.

Starting on day 15, patients began a taper (reduction) of corticosteroid from the starting dose of 10 to 25 mg/d of prednisone (or equivalent) to a target dose of 5 mg/d, which was to be completed on or before day 50. The corticosteroid dose was to be tapered every 1 to 2 weeks, depending on the starting dose, with smaller incremental titrations allowed per the investigator’s judgment, as long as the patient reached the goal dose by day 50. Patients were maintained at the target corticosteroid dose of 5 mg/d (or equivalent) through week 24. Optional titrations in the corticosteroid dose < 5 mg/d could occur after week 16 if the investigator determined further titration to be feasible. Patients who demonstrated acute worsening of sarcoidosis symptoms or who were unable to tolerate the taper were allowed to receive rescue treatment with higher corticosteroid doses per the site investigators’ clinical judgement; on resolution of symptoms, the taper could be reattempted per the investigators’ judgment.

### Outcomes and Statistical Analysis

The primary end point was to evaluate the safety and tolerability of efzofitimod vs placebo in patients with pulmonary sarcoidosis. Adverse events (AEs) were recorded from the date of informed consent and coded using the Medical Dictionary for Regulatory Activities version 24.0. TEAEs were defined as any AE or worsening of an existing condition after initiation of the study drug through 30 days after the last study. The intensity of each AE was rated by the masked investigator using the National Cancer Institute Common Terminology Criteria for Adverse Events version 5.0. Please refer to e-Appendix 1 for grading of AEs. The TEAEs were summarized by frequency of occurrence, number of patients experiencing the event, relationship to study medication, intensity, and seriousness. Patients were monitored closely during study drug infusions, and any AEs that occurred during or within 24 h after study drug administration were captured as infusion-related reactions (IRRs).

For the secondary outcome of potential corticosteroid-sparing effect of efzofitimod, the analysis included the time-adjusted area under the receiver operating characteristic curve from baseline to week 24 for each patient and a corresponding area under the receiver operating characteristic curve for the posttaper period (day 51 through end of study). The time-adjusted area under the receiver operating characteristic curve approximates the average daily corticosteroid dose per patient over the respective period. The development of antidrug antibody and Jo-1 antibodies (antibodies that recognize histidyl-tRNA synthetase) was used to summarize immunogenicity.

Exploratory outcomes evaluated the change from baseline in lung function through week 24. The change in PRO scores from baseline to week 24 also were assessed as follows: SAT, sarcoidosis-specific patient-reported outcomes of impact of disease and response to therapy^21^; KSQ, 29-item questionnaire related to general health (GH) and lung (range, 1-100; higher numbers indicating better health^22^); Leicester Cough Questionnaire, 19-item self-complete QOL measure of chronic cough (range, 3-21; higher numbers indicating better QOL^23^); FAS, 10 fatigue-related questions (range, 10-50; scores of ≥ 22 are considered to represent substantial fatigue^24^); Self-Administered Computerized Baseline and Transitional Dyspnea Indices, graded assessments of changes in the severity of dyspnea at baseline and at subsequent visits (range, 0-12; the lower the score is the worse the severity of dyspnea^25^).

The primary analysis population (safety set) comprised all patients who received any amount of study drug and was based on the actual treatment received. The primary efficacy analysis was the modified intention-to-treat population, defined as all randomized patients who received at least one administration of study drug.

Statistical analyses were performed in an exploratory manner to reflect the phase 1/2 nature of the study. Continuous variables were summarized using descriptive statistics (number, mean ± SD, and median [interquartile range]). Categorical variables were summarized with the number and percentage of patients within each classification. Any calculated *P* values for exploratory variables were analyzed using either the analysis of covariance or a mixed model for repeated measures with the results presented as the difference between active groups and placebo in the least squares mean change.

## Results

### Baseline Characteristics and Patient Disposition

**Figure 1:**
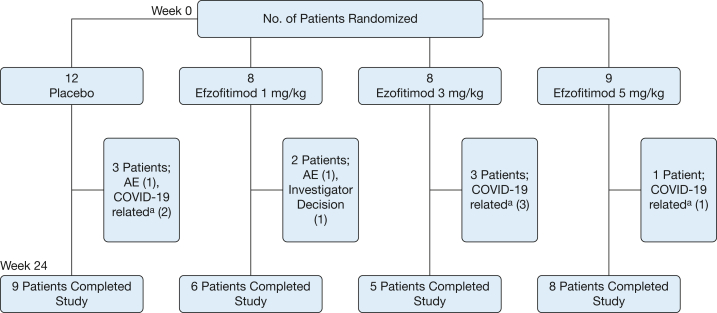
Flow diagram showing patient disposition (Modified Intention-to-Treat Population). ^a^Site closures related to the COVID-19 pandemic. AE = adverse event.

A total of 37 patients were randomized and received at least one dose of study drug: 12 received placebo and eight, eight, and nine patients received the 1 mg/kg, 3 mg/kg, and 5 mg/kg efzofitimod doses, respectively. Nine patients (24%) prematurely discontinued treatment, six because of COVID-19-related restrictions (eg, operational feasibility and site closures), two because of AEs, and one because of investigator decision. Twenty-eight patients (76%) completed the study (Fig 1).

Baseline demographic characteristics generally were well balanced across the treatment groups. The mean ± SD age was 52.4 ± 10.1 years, with 54% women, 62% White, and 38% Black patients. Baseline disease characteristics, including pulmonary function, were similar across treatment groups. Background corticosteroid use generally was comparable across treatment groups; of note, more patients in the placebo group were receiving concomitant immunomodulators compared with the efzofitimod treatment groups. Nearly all patients (36 [97%]) were receiving prednisone (one received methylprednisolone), with a mean ± SD daily steroid dose of 13.2 ± 4.4 mg/d, and 22 patients (59.5%) receiving 10 to < 15 mg/d of prednisone equivalent dose. Demographics and disposition by treatment group are presented in Table 1.

**Table 1:** Baseline Demographics, Disease Characteristics, and Corticosteroid and Immunomodulator Use (Modified Intention-to-Treat Population^a^)

Variable	Placebo (n = 12)	Efzofitimod			All (n = 37)
		1 mg/kg (n = 8)	3 mg/kg (n = 8)	5 mg/kg (n = 9)	
Age, y	52.5 ± 10.2	54.5 ± 11.3	51.8 ± 11.4	50.8 ± 9.8	52.4 ± 10.1
Sex, female	7 (58.3)	4 (50)	4 (50)	5 (55.6)	20 (54.1)
Race					
White	9 (75)	5 (62.5)	6 (75)	3 (33.3)	23 (62.2)
Black	3 (25)	3 (37.5)	2 (25)	6 (66.7)	14 (37.8)
Duration of disease, y					
Median	2.9	5.3	4.3	2.9	4.2
Range	0.5, 10.2	1.5, 19.6	0.6, 15.0	0.5, 28.0	0.5, 28.0
Baseline BDI total score	4.8 ± 2	4.3 ± 1.8	7.6 ± 2.9	6.3 ± 2.5	5.65 ± 2.54
Baseline lung function					
mMRC dyspnea scale score					
1-2	8 (66.7)	3 (37.5)	8 (100)	5 (55.6)	24 (64.9)
3-4	4 (33.3)	5 (62.5)	0	4 (44.4)	13 (35.1)
FEV_1_, % predicted	68.3 ± 20.1	60.4 ± 10.2	77.6 ± 11.1	77.3 ± 19.5	70.8 ± 17.3
FVC, % predicted	77.3 ± 11.5	68.3 ± 9.7	83.8 ± 7.3	83.8 ± 16.6	78.3 ± 12.9
FEV_1_ to FVC ratio	0.7 ± 0.15	0.7 ± 0.08	0.73 ± 0.08	0.72 ± 0.1	0.715 ± 0.11
Dlco, % predicted	61.7 ± 19.7	61.9 ± 21.4	75.5 ± 19.9	54.5 ± 14.1	63.8 ± 19.8
Baseline steroid use					
Prednisone equivalent dose, mg/d^b^	13.3 ± 4.4	11.3 ± 3.5	14.4 ± 6.2	13.9 ± 3.3	13.2 ± 4.4
10 to < 15	7 (58.3)	7 (87.5)	5 (62.5)	3 (33.3)	22 (59.5)
15 to < 20	2 (16.7)	0	0	5 (55.6)	7 (18.9)
≥ 20	3 (25)	1 (12.5)	3 (37.5)	1 (11.1)	8 (21.6)
Baseline immunomodulator use					
Methotrexate	4 (33.3)	2 (25)	0	3 (33.3)	9 (24.3)
Azathioprine	2 (16.7)	0	0	1 (11.1)	3 (8.1)
Hydroxychloroquine	0	1 (12.5)	0	0	1 (2.7)
Leflunomide	0	0	1 (12.5)	0	1 (2.7)
None	6 (50)	5 (62.5)	7 (87.5)	5 (55.6)	23 (62.2)

Data are presented as No. (%) or mean ± SD, unless otherwise indicated. Baseline measurements were defined as the last measurement assessed on or before the first dose date. If multiple measures were obtained on day 1 (eg, vital signs, 12-lead ECG), the last measurement before the first dose was used as baseline. BDI = baseline dyspnea index;Dlco = diffusing capacity of lungs for carbon monoxide; mMRC = modified Medical Research Council.

a All randomized patients who received at least one administration of study drug.

b All steroids were converted to prednisone dose equivalent.

### Safety and Tolerability

No deaths or drug-related serious AEs were observed in the study. Overall, the proportion of patients with an AE was similar between the placebo and efzofitimod treatment groups, with no relationship between AE frequency and increased efzofitimod dose (Table 2). AEs within the respiratory system disorders system organ class were most common and included cough, wheezing, dyspnea, and upper respiratory tract infection (Table 2). These events were not dose dependent, tended to be mild in severity, and did not limit treatment duration. The high incidence of respiratory events across all treatment groups is expected in this patient population and aligns with the underlying disease.

**Table 2:** TEAEs > 15% and Their Relationship to Treatment (Safety Set)

Preferred Term All Causality	Placebo (n = 12)	Efzofitimod		
		1 mg/kg (n = 8)	3 mg/kg (n = 8)	5 mg/kg (n = 9)
Any TEAE	10 (83.3)	8 (100)	7 (87.5)	8 (88.9)
Cough	1 (8.3)	4 (50)	2 (25)	1 (11.1)
Fatigue	0	2 (25)	1 (12.5)	4 (44.4)
Wheezing	0	4 (50)	0	1 (11.1)
AST increased	2 (16.7)	0	0	0
Dizziness	1 (8.3)	1 (12.5)	1 (12.5)	2 (22.2)
Dyspnea	0	0	2 (25)	0
Arthralgia	0	1 (12.5)	2 (25)	0
Headache	1 (8.3)	0	2 (25)	1 (11.1)
Upper respiratory tract infection	1 (8.3)	1 (12.5)	2 (25)	0
Back pain	0	0	2 (25)	0

Data are presented as No. (%). AST = aspartate aminotransferase; TEAE = treatment-emergent adverse event.

During the treatment period, four patients (33%) in the placebo group and four patients (16%) receiving efzofitimod experienced Grade 3 TEAEs. For placebo-treated patients, these events included urticaria, streptococcal sepsis, bradycardia, and worsening of pulmonary sarcoidosis. The relationship between sarcoidosis and study drug was designated as unlikely related, whereas urticaria was considered related to the study drug. One patient in the placebo arm experienced two Grade 3 AEs (bradycardia and worsening pulmonary sarcoidosis), which were considered unlikely to be related to the study drug. In efzofitimod-treated patients, two events occurred at 1 mg/kg (acute cholecystitis and depression) and two events occurred at 5 mg/kg (toothache and myalgia). None of the Grade 3 events reported with efzofitimod were considered possibly related or related. Serious TEAEs were reported in one patients (8.3%) who received placebo and one patient (4%) who received efzofitimod: streptococcal sepsis (placebo) and acute cholecystitis (1 mg/kg efzofitimod).

Two patients discontinued study treatment because of an AE, both of which were assessed as related to the study drug: one patient in the placebo group because of urticaria and one patient in the 1 mg/kg efzofitimod group because of alopecia. One additional patient in the 1 mg/kg efzofitimod group experienced an acute exacerbation of pulmonary sarcoidosis that was considered unrelated to study drug, but resulted in treatment discontinuation based on investigator discretion. Overall, the incidence of patients reporting IRRs was low, with only one patient in the 3 mg/kg efzofitimod group experiencing mild to moderate IRRs on three separate occasions that were considered by the investigator to be related to study drug, but did not require interruption of the infusion. No patients received efzofitimod who showed positive results for antiefzofitimod or antihistidyl-tRNA synthetase antibodies (eg, Jo-1), and no apparent trends were seen within or across treatment groups for vital signs, ECG findings, or blood oxygen saturation levels.

### Corticosteroid Use, Lung Function, and QOL

The average daily dose of corticosteroid at baseline was comparable across treatment arms. All efzofitimod groups showed a lower corticosteroid use through week 24 compared with the placebo group. These reductions seemed to be dose dependent, with the largest percent reduction observed in the 5 mg/kg treatment group, with a 58% decrease from baseline (Table 3) compared with a 46% decrease in placebo, a difference of 12%. Average daily steroid doses through end of study were 6.8 mg, 6.5 mg, and 5.6 mg for the 1 mg/kg, 3 mg/kg, and 5 mg/kg groups, respectively, compared with 7.2 mg for the placebo group, resulting in a baseline-adjusted relative steroid reduction of 5%, 9%, and 22%, respectively. A comparison of adjusted means between placebo and efzofitimod revealed that the highest two efzofitimod treatment groups showed a larger, although statistically nonsignificant, percent decrease from baseline in overall corticosteroid use during the study (–2% for 3 mg/kg and –12% for 5 mg/kg) compared with placebo. Supporting these dose-related trends in corticosteroid reduction, three patients (33%) treated at the highest dose (5 mg/kg) were able to taper off corticosteroid completely and maintain this through the end of the study.

**Table 3:** Corticosteroid^a^ Burden (Modified Intention-to-Treat Population)

Parameter	Placebo (n = 12)	Efzofitimod		
		1 mg/kg (n = 8)	3 mg/kg (n = 8)	5 mg/kg (n = 9)
Baseline prednisone equivalent dose, mg/d	13.3 ± 4.4	11.3 ± 3.5	14.4 ± 6.2	13.9 ± 3.3
Average daily dose, mg^b^	7.2	6.8	6.5	5.6
Change from baseline, %	–45.7 ± 26.7	–41.4 ± 15.9	–48.9 ± 19.7	–58.1 ± 23.4
Difference in adjusted means, %^c^	—	1.2 (–20.0 to 22.4)	–2.3 (–23.1 to 18.5)	–12.3 (–33.1 to 8.5)
Tapered to 0 mg and maintained taper	0	0	0	3 (33.3)

Data are presented as No. (%), mean ± SD, mean, or time-adjusted area under the curve (95% CI).

a Any corticosteroid that was not prednisone was converted to prednisone equivalent dose. All end points use the posttaper period (day 51 to end of dosing).

b Adjusted means from analysis of covariance adjusting for baseline steroid use.

c Time-adjusted area under the curve of percent change from baseline, *P* > .05.

**Figure 2:**
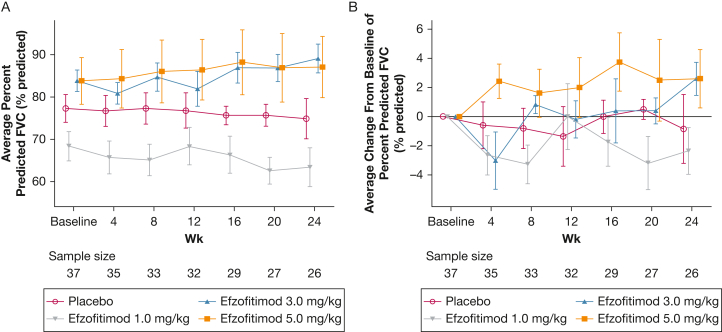
A, B, Line graphs showing change from baseline lung function in the efzofitimod vs the placebo groups (modified intention-to-treat population): absolute FVC % predicted (A) and absolute change from baseline in FVC % predicted (B) from day 1 to the end of study.

Overall, the two highest doses of efzofitimod resulted in improvements in key lung function parameters at week 24 from baseline compared with placebo, consistent with a dose-dependent effect (e-Table 2). Relative to placebo, the effects of 5 mg/kg efzofitimod were observed early (eg, week 4 for FVC % predicted and week 12 for Dlco % predicted) and were maintained across all evaluated time points through week 24. Overall, FVC % predicted declined over the study period with placebo and 1 mg/kg efzofitimod and increased with the higher doses of efzofitimod (Fig 2). Although the improvements in FVC % predicted and Dlco % predicted did not achieve statistical significance at the 5 mg/kg dose level, in part because of the limited sample size, the trend we observed signifies the possibility of biological activity warranting further investigation in a larger population.

Results observed with the PROs evaluated support the hypothesis that the 5 mg/kg dose group may provide a benefit, with trends that occasionally were statistically significant improvements in the mean change from baseline to week 24 for SAT lung, KSQ lung, KSQ GH, and FAS compared with placebo (Table 4); trends in PROs appeared before week 24 (e-Fig 1). After 5 mg/kg efzofitimod, significant improvements were observed before week 24 as follows: SAT lung, statistical improvement observed by week 12 (e-Table 3); KSQ lung, statistical improvement observed by week 8 (e-Table 4); KSQ GH, statistical improvement observed by week 4 and maintained through week 24 (e-Table 5); and trends toward improvement in FAS observed at week 8 (e-Table 6). The changes in other PROs or PRO domains were variable (e-Table 7).

**Table 4:** Change in PROs at Week 24 from Baseline in the Efzofitmod vs Placebo Groups (Modified Intention-to-Treat Population)

PRO Measurement (Adjusted Mean^a^)	1 mg/kg (n = 8)	3 mg/kg (n = 8)	5 mg/kg (n = 9)
SAT lung			
Difference in Least Square Means	4.67	–6.66	–6.42
95% CI	–1.15, 8.19	–12.22, –0.47	–11.7, –1.13
*P* value	.18	**.038**	**.018**
KSQ lung			
Difference in Least Square Means	–6.41	11.29	16.17
95% CI	–20.47, 7.65	–3.39, 25.96	2.49, 29.85
*P* value	.35	.12	**.022**
KSQ general health			
Difference in Least Square Means	–5.1	2.13	18.33
95% CI	–18.52, 8.32	–12.76, 17.01	5.16, 31.49
*P* value	.44	.77	**.008**
FAS total			
Difference in Least Square Means	0.76	–4.78	–7.77
95% CI	–5.09, 6.62	–11.22, 1.65	–13.50, –2.03
*P* value	.79	.14	**.010**

Boldfaced values denote significance *P* < .05. FAS = Fatigue Assessment Scale; KSQ = King’s Sarcoidosis Questionnaire; PRO = patient-reported outcome; SAT = Sarcoidosis Assessment Tool.

a Adjusted means taken from mixed model for repeated measures analysis adjusting for corresponding baseline score.

## Discussion

The results from the present study suggest that efzofitimod is safe and well tolerated in patients with pulmonary sarcoidosis, with no clear dose relationship regarding the incidence of TEAEs. Specifically, no apparent trends overall, within or across efzofitimod treatment groups, were seen regarding change from baseline in clinical laboratory test results, and no notable differences from placebo were observed. No deaths and or discontinuations occurred because of serious AEs in efzofitimod-treated patients. Overall, a low rate of serious or related AEs were noted, with no dose-dependent relationship between AE frequency and increased dose. The nonserious Grade 3 AEs reported after efzofitimod treatment (depression, toothache, and myalgia) were deemed by the investigator to be unlikely to be related to the study drug and did not result in hospitalization or threat of hospitalization. No immunogenicity was reported, as supported by the low incidence of patients with IRRs (n = 1) and the lack of antidrug antibody induction after repeat infusions.

All efzofitimod treatment groups showed a lower corticosteroid use at week 24 compared with the placebo group, which seemed to be dose dependent, with the largest difference observed in the 5 mg/kg treatment group. Four patients were able to taper off prednisone completely (one patient in the placebo group and three patients receiving 5 mg/kg). However, the patient receiving placebo could not be maintained off prednisone for more than 8 weeks because of worsening sarcoidosis and required resumption of prednisone to 10 mg/d. Overall, corticosteroid tapering was possible in all groups, consistent with prior data showing that patients with sarcoidosis in clinical trials can taper corticosteroid successfully over a span of several months.^26^ However, 20% to 74% of patients will experience relapses, with approximately 50% occurring within 6 months of stopping therapy.^27^^,^^28^ Despite the short duration of this trial, we were able to demonstrate numerical differences in tolerance to corticosteroid taper among the study groups, which may suggest biological activity of the medication. Although the magnitude of steroid reduction might be deemed small, steroid toxicity depends on cumulative exposure (daily dose × duration). A 10% decrease in the daily dose may result in a meaningful decrease in the cumulative exposure over 1 year. Indeed, the European Repitatory Society (ERS) considers steroid reduction a critical outcome measure.^2^

Compared with placebo, the highest doses of efzofitimod resulted in improvements in FVC % predicted and Dlco % predicted through week 24, suggesting a dose-dependent effect on lung function. The improvement of FVC % predicted observed in the current study was small, but all patients were receiving baseline antiinflammatory therapy for pulmonary sarcoidosis at the time of study entry. Studies of chronic pulmonary sarcoidosis rarely have demonstrated a significant improvement in FVC % predicted.^2^^,^^29^ The changes in FVC % predicted reported herein were similar to those observed in the treatment arm of a randomized trial of infliximab, in which the mean 24-week improvement was 2.5%.^30^ In that trial, corticosteroids were not tapered. Randomized trials of corticosteroid monotherapy as initial therapy for pulmonary sarcoidosis have found similar improvements in FVC % predicted.^31^^,^^32^

Changes in QOL have been considered a major priority for treatment of sarcoidosis.^33^ QOL end points were exploratory assessments that were not corrected for multiple hypothesis testing. We observed significant improvements at week 24 in PROs, such as SAT lung, KSQ lung, KSQ GH, and FAS, in the 5 mg/kg group. Patients who received 5 mg/kg efzofitimod demonstrated significant improvements in KSQ lung at week 8 and significant improvements in KSQ GH as early as week 4; both of these PROs maintained significance through week 24. The magnitude of change in these PROs at 24 weeks exceeded the minimal clinically important differences (MCIDs). For example, placebo-adjusted KSQ GH improved by 18.3 points with 5 mg/kg efzofitimod vs the MCID of 8 points; for KSQ lung, both the 3 mg/kg (+11.3 points) and 5 mg/kg (+16.2 points) efzofitimod groups exceeded the MCID of 4 points, although only the highest dose was statistically significant. For the SAT lung (MCID estimate, –2.7 points), the change in the 3 mg/kg (–6.5 points) and 5 mg/kg (–6.4 points) groups both reflect meaningful improvements.^21^ The changes in the FAS also exceeded the MCID of 4 points^34^; however, these patients may still have noted some fatigue. The magnitude of these changes exceeds those seen in prior studies evaluating changes in health-related QOL during treatment of pulmonary sarcoidosis.^35^^,^^36^

The major limitation of this study is the small sample size, and as such the results will need to be confirmed in a larger study. Operational site difficulties imposed by the COVID-19 pandemic accounted for most of the dropouts. Because of the size, baseline imbalances were present for several of the key end points and the CIs are fairly wide, which limits our ability to draw firm conclusions. However, statistical analyses when adjusted for baseline value demonstrated that the major end points exhibited directionality in favor of a beneficial effect of efzofitimod, suggesting that a larger sample may solidify the findings. We also acknowledge that statistical adjustment for multiple hypothesis testing and power analysis were not performed because of the exploratory nature of the study. Most patients in this study were receiving > 10 mg of prednisone; therefore, further improvement in FVC % predicted was not likely to be demonstrated. However, the current findings suggest a corticosteroid-sparing effect and improved QOL beyond the MCID. Another limitation is the absence of a rigid corticosteroid tapering protocol based on defined thresholds for measurable physiologic indexes or PROs. Variability in corticosteroid tapering aggressiveness may introduce residual bias in the results, but in general this effect likely would tend to reduce the chances of positive findings, rather than increase them, because the patients were randomized. Allowing some investigator discretion about corticosteroid tapering more accurately mirrors usual practice. It is also not possible to determine whether improvements in QOL scores directly reflect efzofitimod activity or occurred indirectly because efzofitimod allowed a greater reduction of prednisone, which has been associated with worse QOL as measured by the Short-Form 36 (SF-36) and St. George’s Respiratory Questionnaire (SGRQ).^7^ The dose response suggests that the medication itself, rather than a corticosteroid reduction, is more likely to contribute to the observed improvement. Finally, several patients dropped out of the study because of the challenges of clinical trials during the COVID-19 pandemic.

## Interpretation

In patients with pulmonary sarcoidosis, efzofitimod was safe and well tolerated. Exploratory analyses suggest clinically meaningful improvements after 5 mg/kg efzofitimod with corticosteroid use and improvements in lung function and PROs compared with placebo, without increasing the risk of side effects. However, because of the limited number of patients enrolled in this trial, these findings should be considered only as hypothesis generating. These results support further evaluation in prospective trials of efzofitimod in patients with pulmonary sarcoidosis.

## Funding/Support

Supported by 10.13039/100014933aTyr Pharma L. A. M. is supported by the 10.13039/100000002National Institutes of Health [Grants R01HL140357, R01HL142049, and R01HL136681]. P. H. S. S. is supported by the 10.13039/100000002National Institutes of Health [Grants R13HL142300and R01HL131745] and the American Thoracic Society Foundation.

## Financial/Nonfinancial Disclosures

The authors have reported to *CHEST* the following: D. A. C. has received grants from 10.13039/100014468Mallinckrodt Pharmaceuticals, Boehringer Ingelheim, the 10.13039/100015131Foundation for Sarcoidosis Research (FSR), and the Ann Theodore Foundation; serves as a consultant for Roivant Sciences and Boehringer Ingelheim; serves as a member of the adjudication committee for Pliant Therapeutics; and serves as president of the World Association for Sarcoidosis and Other Granulomatous Disorders. S. A. received conference travel support from and is an advisory board member for aTyr Pharma, Inc. C. C. W. H. has received grant funding from Mallinckrodt Pharmaceuticals. L. A. M. has received grants from the FSR, Mallinckrodt Pharmaceuticals, and the University of Cincinnati (Mallinckrodt Pharmaceuticals Foundation Grant) and serves on the scientific advisory board for FSR and the global advisory board for aTyr Pharma, Inc. O. N. O. has received travel support from aTyr Pharma, Inc., was provided equipment for conducting the current study, and serves on the scientific advisory board for FSR. P. H. S. S. has received grants the FSR, aTyr Pharma, Inc., and Novartis. S. S. and G. W. are employed by and own stock in aTyr Pharma, Inc. N. K. has received payments from aTyr Pharma, Inc., for consultancy on the statistical analysis and interpretation of data, payments from Savara Pharmaceuticals for consultancy on the design and analysis of clinical trials in respiratory diseases, and previously held stock in Roche. R. B. has received grants from 10.13039/100004326Bayer, 10.13039/100004328Genentech, Mallinckrodt Pharmaceuticals, the FSR, and Actelion; consulting fees from Mallinckrodt Pharmaceuticals, Meitheal Pharmaceuticals, Actelion, and Kinevant; and payment for speaker bureaus from Mallinckrodt Pharmaceuticals, United Therapeutics, and Boehringer Ingelheim. None declared (J. B., W. E. J., L. T. M., and N. J. S.).

## Supplementary Data

e-Online Data
