# Supplementary material for: Efzofitimod for the Treatment of Pulmonary Sarcoidosis
Source: Chest. 2022 Nov 8;163(4):881–90. doi: 10.1016/j.chest.2022.10.037 (PMC10258437; doi:10.1016/j.chest.2022.10.037)
Supplement: e-Online Data [file mmc1.docx]

Online Data Supplement

Methods

**Inclusion and Exclusion Criteria**

Inclusion Criteria:

The following inclusion criteria must be met for a patient to be eligible for inclusion in the study:

1. Male or female patients aged ≥18 to ≤75 years inclusive at time of informed consent
2. Diagnosis of pulmonary sarcoidosis for ≥6 months (cutaneous and ocular involvement allowed), defined as:

- Histologically proven diagnosis of sarcoidosis by bronchoscopy, biopsy (any organ) or bronchioalveolar lavage.
- Evidence of parenchymal lung involvement by historical radiological evidence (eg, computed tomography [CT], magnetic resonance imaging [MRI], 18F-FDG PET/CT or chest X-ray) or on the Screening 18F-FDG PET/CT.

1. Must have symptomatic and/or active pulmonary sarcoidosis as evidence by:

- Clinical findings of dyspnea, as indicated by a Modified Medical Research Council (MRC) Dyspnea Scale grade of at least 1; and
- FVC% predicted ≥50%.

1. Must be receiving treatment with 10 to 25 mg/day of oral prednisone (or oral equivalent; eg, methylprednisolone), at a stable dose for ≥4 weeks prior to Day 1, and be determined by the Investigator to be capable of undergoing the protocol-specified steroid taper regimen.

- Treatment with one oral immunomodulatory therapy (eg, methotrexate, azathioprine, hydroxychloroquine) at a stable dose for ≥1 month prior to Day 1 is allowed but not required. The dose of this therapy should remain constant throughout the study.

1. Body weight ≥45 kg and <160 kg.
2. Female patients may be of childbearing potential or of non-childbearing potential (either surgically sterilized or at least 1 year postmenopausal (confirmed by amenorrhea duration of at least 12 months and serum follicle-stimulating hormone [FSH] ≥30 mIU/mL).

- Female patients of childbearing potential must be non-pregnant and non-lactating, and have a negative pregnancy test at Screening (serum) and at Day 1 (urine) prior to first study drug infusion.
- Additionally, female patients of childbearing potential must be willing to use highly-effective contraception (see Section 7.1.6.4) from Screening until 90 days after the last follow-up visit.

1. Male patients, if not infertile or surgically sterilized, must agree to use highly-effective contraception (see Section 7.1.6.4) and not donate sperm from Screening until 90 days after the last follow-up visit.
2. Provision of written informed consent.
3. Be able to communicate well with the Investigator and site personnel, and agree to comply with all study procedures and requirements.

Exclusion Criteria:

A patient who meets any of the following exclusion criteria will not be eligible for inclusion in the study:

1. Current disease presentation consistent with Lofgren’s syndrome (ie, presence of the triad of erythema nodosum, bilateral hilar lymphadenopathy on chest X-ray, and joint pain).
2. History of severe allergic or anaphylactic reactions to therapeutic proteins, or known sensitivity to ATYR1923 or to its inactive components (L-histidine, sodium chloride, sucrose, L-methionine, and polysorbate-20).
3. Treatment (within 4 months of Day 1) with biological immunomodulators such as tumor necrosis factor-alpha (TNF-α) inhibitors (eg, infliximab, adalimumab).
4. Current evidence of clinically significant cardiovascular, hepatic, renal, hematological, metabolic, or gastrointestinal disease, or has a condition that requires other treatment, may not allow safe participation, or which in the opinion of the Investigator should preclude the patient’s participation in the clinical study.
5. Clinically significant pulmonary hypertension requiring vasodilator treatment.
6. Any history of tuberculosis, or evidence of active systemic non-tuberculous fungal or mycobacterial infection within 1 year of Screening.
7. History of clinically significant cardiac, neurological, gastrointestinal, and/or renal manifestations of their sarcoidosis.
8. Active or history of malignancy within the last 5 years, with the exception of resected basal cell carcinoma, squamous cell carcinoma of the skin, or effectively managed cervical carcinoma.
9. Major surgery within 3 months prior to Day 1 or anticipated surgery during the study.
10. Any condition that necessitated hospitalization within the 3 months prior to Day 1 or is likely to require so during the study.
11. Participation in another clinical study of an investigational agent or device within 3 months (small molecules) / 6 months (biologics) or 5 half-lives (if known) of the agent, whichever is longer.
12. History of or positive results of screening for hepatitis B (hepatitis B surface antigen [HBsAg]), hepatitis C (anti-hepatitis C virus [HCV] antibodies [Ab]) or human immunodeficiency virus (HIV) (HIV Ab type 1 and 2).
13. Is an active, heavy smoker of tobacco/nicotine-containing products (defined as >20 cigarettes/day or e-cigarette equivalent).
14. Active substance abuse (drugs or alcohol) or history of substance abuse within the 12 months prior to Screening.
15. Clinically significant abnormalities in the Screening physical examination, vital signs, ECG, or clinical laboratory test results that, in the opinion of the Investigator and Medical Monitor should preclude the patient’s participation in the clinical study.
16. Patient has received a live vaccination within 8 weeks before Day 1 or inoculation with a live vaccine is planned during study participation.
17. Jo-1 Ab levels >7 U/mL at Screening, or past history of Jo-1 Ab positivity.
18. Any other condition or circumstance that, in the opinion of the investigator, would be likely to prevent adequate compliance with the study protocol.
19. Significant and/or acute illness (eg, change in pulmonary status, infection requiring antibiotics) within 5 days prior to (the first) drug administration that may impact safety assessments, in the opinion of the Investigator.

**Grading of Adverse Events**

The intensity of each AE was rated by the Investigator using the National Cancer Institute Common Terminology Criteria for Adverse Events (NCI CTCAE), version 5.0. AEs not listed on the NCI CTCAE are to be rated by the Investigator as “mild (Grade 1)”, “moderate (Grade 2)”, “severe” (Grade 3), “life-threatening” (Grade 4), or “fatal” (Grade 5). In addition to patient symptoms, clinically significant new findings on physical exam, 12-lead ECG, vital sign assessment or clinical laboratory reports were to be reported as AEs. The number and percent of patients with any TEAEs as well as number of TEAEs were summarized by system organ class and preferred term by treatment and overall.

e-Table 1: Schedule of Assessments

| **Study Period** | **Screen** | **Treatment** | | | | | | | | | | | | | **EOS** |
| --- | --- | --- | --- | --- | --- | --- | --- | --- | --- | --- | --- | --- | --- | --- | --- |
| **Visit** |  | **1** | **1a** | **2** | **2a** | **3** | **3a, 3b, 3c** | **4** | **4a** | **5** | **5a** | **6** | **6a** | **7** |  |
| **Study Day** | **-28 to - 1** | **D1** | **W1/ D8** | **W2/** **D15** | **W3/** **D22** | **W4/** **D29** | **W5, 6, 7** | **W8/** **D57** | **W10/** **D71** | **W12/** **D85** | **W14/** **D99** | **W16/** **D113** | **W18/** **D127** | **W20/** **D141** | **WK24/D169** |
| **Visit Window (Days)** | **-** | **-** | **-** | **±2** | **±3** | **±3** | **±2** | **±3** | **±3** | **±3** | **±3** | **±3** | **±3** | **±3** | **±3** |
| Written informed consent | X |  |  |  |  |  |  |  |  |  |  |  |  |  |  |
| Telephone Contact |  |  | X |  | X |  | X |  | X |  | X |  | X |  |  |
| Eligibility check | X | X |  |  |  |  |  |  |  |  |  |  |  |  |  |
| Demographics | X |  |  |  |  |  |  |  |  |  |  |  |  |  |  |
| Medical history | X |  |  |  |  |  |  |  |  |  |  |  |  |  |  |
| Height & weight^1^ | X | X |  |  |  | X |  | X |  | X |  | X |  | X |  |
| Modified MRC Dyspnea Scale | X |  |  |  |  |  |  |  |  |  |  |  |  |  |  |
| Physical examination^2^ | X |  |  |  |  |  |  |  |  | X |  |  |  |  | X |
| Vital signs^3^ | X | X |  | X |  | X |  | X |  | X |  | X |  | X | X |
| Pulse oximetry^4^ | X | X |  |  |  | X |  | X |  | X |  | X |  | X | X |
| 12-lead ECG^5^ | X | X^5^ |  |  |  | X |  | X |  | X^5^ |  | X |  | X^5^ | X |
| Pulmonary Function Tests^6^ | X | X |  |  |  | X |  | X |  | X |  | X |  | X | X |
| DL_CO_^7^ |  | X |  |  |  |  |  |  |  | X |  |  |  | X | X |
| Pregnancy test (females only)^8^ | X (serum) | X |  |  |  |  |  | X |  |  |  | X |  |  | X (serum) |
| Serum FSH^9^ | X |  |  |  |  |  |  |  |  |  |  |  |  |  |  |
| Rheumatoid factor |  | X |  |  |  |  |  |  |  |  |  |  |  |  |  |
| Jo-1 antibody (serum) | X |  |  | X |  | X |  | X |  | X |  | X |  | X | X |
| ADA sampling (serum) for anti-ATYR1923 antibodies^17^ | X |  |  | X |  | X |  | X |  | X |  | X |  | X | X |
| Safety laboratory testing (hematology, clinical chemistry) | X | X |  | X |  | X |  | X |  | X |  | X |  | X | X |
| Urinalysis^10^ | X | X |  | X |  | X |  | X |  | X |  | X |  | X | X |
| Coagulation laboratory testing (PT, INR, PTT) | X |  |  |  |  | X |  | X |  | X |  | X |  | X | X |
| Serology (HBsAg, anti-HCV, and anti-HIV 1/2 tests) | X |  |  |  |  |  |  |  |  |  |  |  |  |  |  |
| Serum complement, serum tryptase, and IgE^11^ |  | X |  |  |  |  |  |  |  |  |  |  |  |  |  |
| Plasma complement^11^ |  | X |  |  |  |  |  |  |  |  |  |  |  |  |  |
| ^18^F-FDG-PET/CT (optional)^12^ | X |  |  |  |  |  |  |  |  |  |  | X |  |  |  |
| Skin lesion visual assessments (if applicable)^13^ | X |  |  | X |  | X |  | X |  | X |  | X |  | X |  |
| Skin lesion biopsy (optional for patients at select sites)^14^ | X |  |  |  |  |  |  |  |  | X |  |  |  |  |  |
| AE assessment/Concomitant medications |  | X | X | X | X | X | X | X | X | X | X | X | X | X | X |
| Randomization^15^ |  | X |  |  |  |  |  |  |  |  |  |  |  |  |  |
| King’s Sarcoidosis Questionnaire |  | X |  |  |  | X |  | X |  | X |  | X |  | X | X |
| Leicester Cough Questionnaire |  | X |  |  |  | X |  | X |  | X |  | X |  | X | X |
| Baseline/Transitional Dyspnea Indices |  | X |  |  |  | X |  | X |  | X |  | X |  | X | X |
| Fatigue Assessment Scale |  | X |  |  |  | X |  | X |  | X |  | X |  | X | X |
| Sarcoidosis Assessment Tool |  | X |  |  |  | X |  | X |  | X |  | X |  | X | X |
| Blood sampling (serum) for ATYR1923 PK^16^ |  | X^16^ |  | X |  | X |  | X |  | X |  | X |  | X^16^ | X |
| PBMC collection^18^ |  | X |  |  |  |  |  |  |  | X |  |  |  |  | X |
| Serum biomarkers^19^ |  | X |  |  |  |  |  |  |  | X |  |  |  | X | X |
| Infusion site examination^20^ |  | X |  |  |  | X |  | X |  | X |  | X |  | X |  |
| Study drug administration |  | X |  |  |  | X |  | X |  | X |  | X |  | X |  |
| OCS Taper^21^ |  |  |  | X |  |  |  |  |  |  |  |  |  |  |  |

ADA = anti‑drug antibodies; AE = adverse event; BMI = body mass index; D = Day; ECG = electrocardiogram; EOI = end of infusion; EOS = End-of-Study; ET= Early Termination; FSH = follicle stimulating hormone; HBsAg = hepatitis B surface antigen; HCV = hepatitis C virus; HIV = human immunodeficiency virus; HRCT = high-resolution computed tomography; INR = international normalized ratio; OCS = Oral Corticosteroid; PBMC = peripheral blood mononuclear cell; PD = pharmacodynamics; PET = positron emission tomography; PK = pharmacokinetics; PT = prothrombin time; PTT = partial thromboplastin time; SOI=start of infusion; W = week(s).

On dosing days, all assessments will be performed pre-dose unless otherwise specified.

1. **Height** only at Screening.

2. P**hysical examination**. Full physical examinations are to be obtained at Screening, Week 12 and 24, abbreviated symptom-directed physical examination may be completed at other visits if needed.

3. **Vital signs** are to be obtained at every visit. On study drug administration days, vital signs are to be measured pre-infusion and at 15 and 30 minutes (±5 minutes) and at 1, 2, and 4 hours (±15 minutes) after the start of infusion (SOI). Vital signs are to be measured before blood sample collection. Vital signs will include blood pressure (systolic and diastolic, recorded after lying supine for 5 min), heart rate, respiratory rate. Temperature is to be obtained at Screening and on dosing days at pre-dose, 60 minutes (±15 minutes) after the SOI, and again at 4 hours (±15 minutes) after the SOI.

4. **Pulse oximetry:** continuous pulse oximetry is to be measured on dosing days from 5 minutes pre-dose until EOI, and recorded at the same time points as vital signs. On non-dosing days pulse oximetry is to be obtained with vital sign assessments.

5. **12-lead ECG** is to be obtained at Screening, within 1-hour pre-dose on dosing days. At Day 1 and Weeks 12 and 20, an ECG is to be obtained at 4 hours (±30 minutes) after SOI. The ECG is to be obtained after patients have been lying supine for 5 minutes.

6. **Pulmonary Function Testing** is to be performed using the same spirometer throughout the study. Parameters include forced vital capacity (FVC), forced expiratory volume in 1 second (FEV_1_), FEV_1_/FVC ratio at all specified visits. A minimum of 3 efforts should be obtained that meet the acceptance criteria of the American Thoracic Society or European Respiratory Society.

7. **DL_CO_** measurements *should* be obtained after patients have been sitting quietly for 5 minutes.

8. **Pregnancy** serum tests are to be performed on all females at Screening and at Week 24/EOS. A urine pregnancy test is to be performed at other time points indicated.

9. **FSH** only required for all female patients.

10. **Urinalysis** (semi-quantitative by dipstick): microscopy is to be performed if indicated by an abnormal and clinically significant result.

11. **Serum Complement, Tryptase/Plasma Complement, and IgE** are to be collected at Day 1 pre-dose and again if an infusion related reaction (IRR) occurs.

12. **^18^F-FDG-PET/CT scans are optional**; patients who elect to participate agree to have imaging performed within 4 weeks prior to Day 1, and within ±5 days of Week 16 or if early termination prior to Week 16.

13. **Skin lesion evaluation:** To be completed for patients with skin lesions present. Skin lesions will be evaluated by: Skin Physician Global Assessment (SPGA), body surface Area assessment, the Sarcoidosis Activity and Severity Index (SASI).

14. **Skin lesion biopsy:** Optional for patients with skin lesions present. A non-target lesion (ie, a lesion that is not being assessed by SASI over time) is to be selected by the Investigator for biopsy during Screening and confirmed by the patient that it had been identified by his or her private dermatologist as cutaneous sarcoidosis. Skin lesion biopsy may be obtained within 4 weeks prior to Day 1 for patients who are otherwise deemed eligible for the study and within ±5 days of Week 12 or if early termination prior to Week 12.

15. **Randomization** is to be performed within 0-3 days prior to Day 1 or on the day of dosing.

16. **ATYR1923 serum PK** samples are to be collected pre-dose on dosing days. On Day 1 and Week 20 visits, PK samples are also collected post start of infusion at: 1 hr (just prior to [i.e., within 0-10 minutes] EOI), and at any single time point between 4-6 hours.

17. **ADA** samples are to be collected pre‑dose when obtained on dosing days.

18. **Blood for PBMC assessments** will be collected pre-dose when obtained on dosing days.

19. **Serum for biomarkers** are to be collected pre-dose when obtained on dosing days.

20. **Infusion site examination:** the IV infusion site should be examined at 0.5 and 1.5 hours (+10 minutes) after the end of infusion.

21. **OCS Taper:** OCS taper to start at Day 15 (Week 2) and continue through Day 50 (Week 7) per the ATYR1923-C-002 OCS Taper Guideline.

e-Table 2: Change from Baseline in Lung Function Efzofitimod vs. Placebo – (modified Intention-to-Treat population)

|  | efzofitimod | | |
| --- | --- | --- | --- |
|  | 1 mg/kg n = 8 | 3 mg/kg n = 8 | 5 mg/kg n = 9 |
| **FVC%** | | | |
| Week 4 Active vs. Placebo |  |  |  |
| Difference in Adjusted Means  (95% CI) | -1.26  (-5.66, 3.15) | -2.98  (-7.35, 1.38) | 2.40  (-2.37, 7.18) |
| p-value | p = 0.58 | p = 0.18 | p = 0.32 |
| Week 8 Active vs. Placebo |  |  |  |
| Difference in Adjusted Means  (95% CI) | -1.53  (-6.01, 2.95) | 0.89  (-3.65, 5.43) | 1.88  (-2.56, 6.32) |
| p-value | p = 0.50 | p = 0.70 | p = 0.41 |
| Week 12 Active vs. Placebo |  |  |  |
| Difference in Adjusted Means  (95% CI) | 2.23  (-2.34, 6.79) | 1.54  (-3.89, 6.97) | 2.69  (-2.02, 7.4) |
| p-value | p = 0.34 | p = 0.58 | p = 0.26 |
| Week 16 Active vs. Placebo |  |  |  |
| Difference in Adjusted Means  (95% CI) | -1.23  (-5.9, 3.4) | 0.06  (-4.9, 5.01) | 3.23  (-1.57, 8.03) |
| p-value | p = 0.61 | p = 0.98 | p = 0.19 |
| Week 20 Active vs. Placebo |  |  |  |
| Difference in Adjusted Means  (95% CI)  p-value | -1.24  (-6.04, 3.6)  p = 0.61 | 1.7  (-3.4, 6.8)  p = 0.51 | 3.46  (-1.59, 8.51)  p = 0.18 |
| Week 24 Active vs. Placebo |  |  |  |
| Difference in Adjusted Means  (95% CI)  p-value | -0.08  (-4.92, 4.76)  p = 0.97 | 2.8  (-2.65, 8.26)  p = 0.31 | 3.3  (-1.9, 8.51)  p = 0.21 |
| **DLco%** | | | |
| Week 12 Active vs. Placebo |  |  |  |
| Difference in Adjusted Means  (95% CI) | 0.49  (-8.96, 9.94) | 6.47  (-4.88, 17.82) | 5.64  (-3.78, 15.06) |
| p-value | p = 0.92 | p = 0.26 | p = 0.24 |
| Week 20 Active vs. Placebo |  |  |  |
| Difference in Adjusted Means  (95% CI) | -0.96  (-11.94, 10.02) | 3.37  (-8.35, 15.1) | 2.35  (-8.13, 12.84) |
| p-value | p = 0.86 | p = 0.57 | p = 0.65 |
| Week 24 Active vs. Placebo |  |  |  |
| Difference in Adjusted Means  (95% CI) | -0.04  (-11.27, 11.19) | 2.96  (-8.87, 14.8) | 7.46  (-3.08, 18.0) |
| p-value | p = 0.99 | p = 0.62 | p = 0.17 |

CI = confidence interval; DLco% = percent-predicted diffusion capacity of the lungs; FVC% = precent-predicted forced vital capacity

* Adjusted Means taken from MMRM analysis adjusting for baseline value of the corresponding lung function parameter on scheduled visits (FVC%: Baseline, W4, 8, 12, 16, 20, 24; (DLco%: Baseline, W12, 20 and 24)

**e-Table 3: Change in SAT-L from Baseline Efzofitimod vs. Placebo - (modified Intention-to-Treat population)**

|  | efzofitimod | | |
| --- | --- | --- | --- |
|  | 1 mg/kg n = 8 | 3 mg/kg n = 8 | 5 mg/kg n = 9 |
| **SAT-L** | | | |
| Week 4 Active vs. Placebo |  |  |  |
| Difference in Adjusted Means  (95% CI) | -1.87  (-7.08, 3.34) | -0.87  (-6.5, 4.77) | -3.82  (-8.83, 1.19) |
| p-value | p = 0.48 | p = 0.76 | p = 0.13 |
| Week 8 Active vs. Placebo |  |  |  |
| Difference in Adjusted Means  (95% CI) | 0.84  (-4.27, 6.05) | -1.09  (-6.88, 4.69) | -1.55  (-6.56, 3.47) |
| p-value | p = 0.75 | p = 0.71 | p = 0.54 |
| Week 12 Active vs. Placebo |  |  |  |
| Difference in Adjusted Means  (95% CI) | -0.18  (-5.39, 5.03) | -5.34  (-11.12, 0.43) | -5.19  (-10.2, -0.17) |
| p-value | p = 0.94 | p = 0.07 | **p = 0.043** |
| Week 16 Active vs. Placebo |  |  |  |
| Difference in Adjusted Means  (95% CI) | 2.84  (-2.6, 8.28) | -4.23  (-10.12, 1.66) | -5.38  (-10.51, -0.25) |
| p-value | p = 0.30 | p = 0.16 | **p = 0.040** |
| Week 20 Active vs. Placebo |  |  |  |
| Difference in Adjusted Means  (95% CI)  p-value | 2.69  (-2.82, 8.19)  p = 0.33 | -6.27  (-12.22, -0.31)  **p = 0.039** | -4.29  (-9.49, 0.9)  p = 0.10 |
| Week 24 Active vs. Placebo |  |  |  |
| Difference in Adjusted Means  (95% CI)  p-value | 4.44  (-1.15, 10.03)  p = 0.12 | -6.49  (-12.52, -0.47)  **p = 0.035** | -6.42  (-11.7, -1.13)  **p = 0.018** |

SAT-L= Sarcoidosis Assessment Tool-Lung

Adjusted Means taken from MMRM analysis adjusting for corresponding baseline score

**e-Table 4: Change in KSQ-L from Baseline Efzofitimod vs. Placebo - (modified Intention-to-Treat population)**

|  | efzofitimod | | |
| --- | --- | --- | --- |
|  | 1 mg/kg n = 8 | 3 mg/kg n = 8 | 5 mg/kg n = 9 |
| **KSQ-L** | | | |
| Week 4 Active vs. Placebo |  |  |  |
| Difference in Adjusted Means  (95% CI) | -2.68  (-8.97, 3.61) | 1.04  (-5.73, 7.82) | 5.06  (-1.19, 11.3) |
| p-value | p = 0.39 | p = 0.76 | p = 0.11 |
| Week 8 Active vs. Placebo |  |  |  |
| Difference in Adjusted Means  (95% CI) | -2.34  (-10.13, 5.46) | 0.81  (-7.93, 9.55) | 11.46  (3.7, 19.22) |
| p-value | p = 0.55 | p = 0.85 | **p = 0.005** |
| Week 12 Active vs. Placebo |  |  |  |
| Difference in Adjusted Means  (95% CI) | -4.47  (-15.55, 6.62) | 4.26  (-7.47, 15.99) | 17.02  (5.95, 28.09) |
| p-value | p = 0.42 | p = 0.47 | **p = 0.004** |
| Week 16 Active vs. Placebo |  |  |  |
| Difference in Adjusted Means  (95% CI) | -5.47  (-16.76, 5.81) | 8.01  (-4.08, 20.09) | 14.72  (3.56, 25.88) |
| p-value | p = 0.33 | p = 0.19 | **p = 0.012** |
| Week 20 Active vs. Placebo |  |  |  |
| Difference in Adjusted Means  (95% CI)  p-value | -2.02  (-17.36, 13.33)  p = 0.79 | 9.75  (-5.28, 24.79)  p = 0.19 | 15.93  (1.09, 30.78)  **p = 0.036** |
| Week 24 Active vs. Placebo |  |  |  |
| Difference in Adjusted Means  (95% CI)  p-value | -6.41  (-20.47, 7.65)  p = 0.35 | 11.29  (-3.39, 25.96)  p = 0.12 | 16.17  (2.49, 29.85)  **p = 0.022** |

KSQ-L = King’s Sarcoidosis Questionnaire – Lung Subscore

Adjusted Means taken from MMRM analysis adjusting for corresponding baseline score

**e-Table 5: Change in KSQ-GH from Baseline Efzofitimod vs. Placebo - (modified Intention-to-Treat population)**

|  | efzofitimod | | |
| --- | --- | --- | --- |
|  | 1 mg/kg n = 8 | 3 mg/kg n = 8 | 5 mg/kg n = 9 |
| **KSQ-GH** | | | |
| Week 4 Active vs. Placebo |  |  |  |
| Difference in Adjusted Means  (95% CI) | -1.45  (-9.64, 6.74) | 1.47  (-7.26, 10.21) | 10.78  (2.4, 19.17) |
| p-value | p = 0.72 | p = 0.73 | **p = 0.0133** |
| Week 8 Active vs. Placebo |  |  |  |
| Difference in Adjusted Means  (95% CI) | -0.41  (-8.14, 7.32) | 0.45  (-8.3, 9.3) | 12.7  (4.74, 20.65) |
| p-value | p = 0.91 | p = 0.92 | **p = 0.003** |
| Week 12 Active vs. Placebo |  |  |  |
| Difference in Adjusted Means  (95% CI) | -1.9  (-12.73, 8.93) | 1.66  (-9.98, 13.31) | 17.85  (6.86, 28.83) |
| p-value | p = 0.72 | p = 0.77 | **p = 0.002** |
| Week 16 Active vs. Placebo |  |  |  |
| Difference in Adjusted Means  (95% CI) | -3.99  (16.45, 8.46) | 7.85  (-5.63, 21.33) | 18.5  (6.15, 30.84) |
| p-value | p = 0.52 | p = 0.25 | **p = 0.005** |
| Week 20 Active vs. Placebo |  |  |  |
| Difference in Adjusted Means  (95% CI)  p-value | -2.01  (-17.44, 13.42)  p = 0.79 | 0.89  (-14.69, 16.46)  p = 0.91 | 19.9  (4.88, 34.92)  **p = 0.011** |
| Week 24 Active vs. Placebo |  |  |  |
| Difference in Adjusted Means  (95% CI)  p-value | -5.1  (-18.52, 8.32)  p = 0.44 | 2.13  (-12.76, 17.01)  p = 0.77 | 18.33  (5.16, 31.49)  **p = 0.008** |

KSQ-GH = King’s Sarcoidosis Questionnaire – General Health (GH) Status Subscore

Adjusted Means taken from MMRM analysis adjusting for corresponding baseline score

**e-Table 6: Change in FAS-total from Baseline Efzofitimod vs. Placebo - (modified Intention-to-Treat population)**

|  | efzofitimod | | |
| --- | --- | --- | --- |
|  | 1 mg/kg n = 8 | 3 mg/kg n = 8 | 5 mg/kg n = 9 |
| **FAS - Total** | | | |
| Week 4 Active vs. Placebo |  |  |  |
| Difference in Adjusted Means  (95% CI) | 1.04  (-2.53, 4.6) | 0.36  (-3.47, 4.18) | -2.08  (-5.75, 1.58) |
| p-value | p = 0.56 | p = 0.85 | p = 0.26 |
| Week 8 Active vs. Placebo |  |  |  |
| Difference in Adjusted Means  (95% CI) | 0.64  (-3.53, 4.81) | -0.60  (-5.08, 3.87) | -5.23  (-9.49, -0.97) |
| p-value | p = 0.76 | p = 0.79 | **p = 0.018** |
| Week 12 Active vs. Placebo |  |  |  |
| Difference in Adjusted Means  (95% CI) | 5.33  (0.86, 9.8) | 0.71  (-4.26, 5.69) | -3.91  (-8.46, 0.64) |
| p-value | p = 0.021 | p = 0.77 | p = 0.09 |
| Week 16 Active vs. Placebo |  |  |  |
| Difference in Adjusted Means  (95% CI) | 4.45  (-1.2, 10.1) | -1.19  (-6.98, 4.59) | -4.31  (-9.89, 1.27) |
| p-value | p = 0.12 | p = 0.68 | p = 0.13 |
| Week 20 Active vs. Placebo |  |  |  |
| Difference in Adjusted Means  (95% CI)  p-value | 2.12  (-4.24, 8.48)  p = 0.51 | -1.81  (-8.22, 4.6)  p = 0.57 | -4.47  (-10.63, 1.7)  p = 0.15 |
| Week 24 Active vs. Placebo |  |  |  |
| Difference in Adjusted Means  (95% CI)  p-value | 0.76  (-5.09, 6.62)  p = 0.79 | -4.78  (-11.22, 1.65)  p = 0.14 | -7.77  (-13.5, -2.03)  **p = 0.010** |

FAS = Fatigue Assessment Scale – Total Score

Adjusted Means taken from MMRM analysis adjusting for corresponding baseline score

e-Table 7: Change in Patient Reports Outcomes (PROs) at Week 24 from Baseline Efzofitimod Treatment vs. Placebo – (modified Intention-to-Treat population)

| PRO Measurement (Adjusted Mean*) | 1 mg/kg  (N = 8) | 3 mg/kg  (N = 8) | 5 mg/kg  (N = 9) |
| --- | --- | --- | --- |
| **LCQ-Total** | -3.49 | 2.98 | 2.05 |
|  | (-6.18, -0.79) | (0.13, 5.83) | (-0.58, 4.69) |
|  | **p = 0.01** | **p = 0.04** | p = 0.12 |
| **LCQ-Psychological** | -1.04 | 0.98 | 1.15 |
|  | (-2.22, 0.13) | (-0.26, 2.23) | (0.01, 2.30) |
|  | p = 0.08 | p = 0.12 | **p = 0.05** |
| **SAT-Satisfaction with Roles and Activities** | -1.41 | 6.50 | 7.6 |
|  | (-9.0, 6.17) | (-1.79, 14.78) | (-0.02, 15.2) |
|  | p = 0.71 | p = 0.12 | **p = 0.05** |
| **SAT-Fatigue** | 2.52 | -5.96 | -10.76 |
|  | (-4.18, 9.23) | (-13.14, 1.22) | (-17.76, -3.95) |
|  | p = 0.46 | p = 0.10 | **p = 0.002** |
| **SAT-Physical Functioning** | 0.44 | 6.21 | 3.93 |
|  | (-4.47, 5.34) | (0.78, 11.63) | (-0.87, 8.73) |
|  | p = 0.86 | **p = 0.03** | p = 0.11 |
| **TDI-Change in Functional Impairment** | -0.8 | 1.87 | 2.34 |
|  | (-4.55, 2.95) | (-1.93, 5.67) | (-1.20, 5.88) |
|  | p = 0.66 | p = 0.32 | p = 0.19 |
| **FAS-Physical Fatigue** | -0.09 | -3.13 | -4.19 |
|  | (-3.83, 3.64) | (-7.24, 0.97) | (-7.92, -0.46) |
|  | p = 0.96 | p = 0.13 | **p = 0.03** |

FAS = Fatigue Assessment Scale; LCQ = Leicester Cough Questionnaire; SAT = Sarcoidosis Assessment Tool; TDI = Transition Dyspnea Index

Adjusted Means taken from MMRM analysis adjusting for corresponding baseline score

e-Figure 1: Change in Patient Reports Outcomes (PROs) at Week 24 from Baseline Active Treatment vs. Placebo – (modified Intention-to-Treat population)


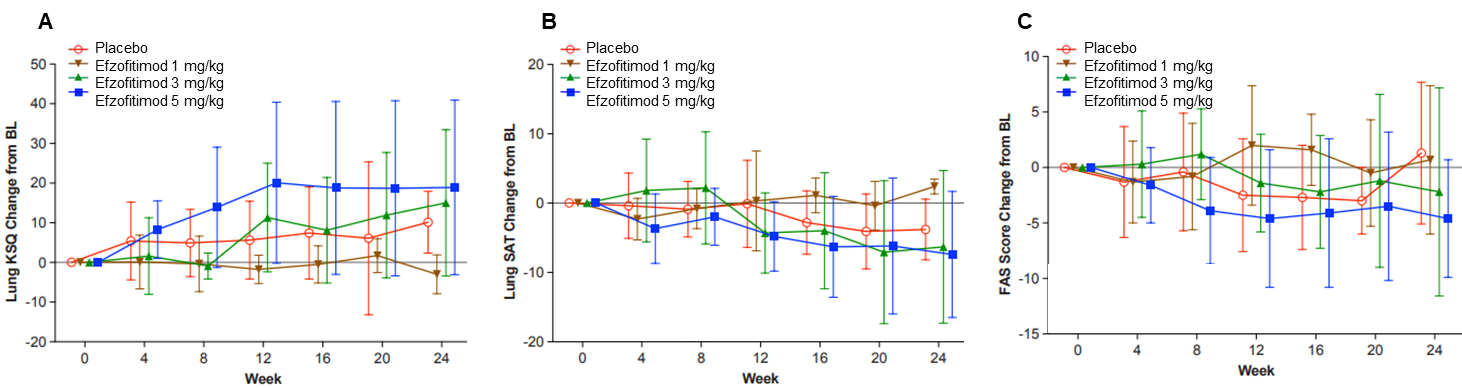


Figure E1: Change in patient reported outcomes (PROs) presented as change from baseline x treatment week for Lung KSQ (A), Lung-SAT (B), and FAS-Total. All data is represented as mean(SD).
